# Anti-Inflammatory Effects of *Amomum villosum* Extract on Dextran Sodium Sulfate-Induced Colitis in Mice

**DOI:** 10.3390/cimb47060389

**Published:** 2025-05-23

**Authors:** Han-Byeol Choi, Ryeo Won Kim, Gi-Sang Bae, Ji Hun Jang, Ye-Seul Kim, Byung Ouk Park, Kang-Beom Kwon

**Affiliations:** 1 Department of Physiology, College of Korean Medicine, Wonkwang University, Iksan 54538, Republic of Korea; hanthf.2@daum.net (H.-B.C.); kys5207@hanamil.net (Y.-S.K.); bopark1@gmail.com (B.O.P.); 2 Ilwonbio Co., Ltd., Iksan 54538, Republic of Korea; aroma9022@gmail.com (R.W.K.); fkzmflahtk@gmail.com (J.H.J.); 3 Department of Pharmacology, School of Korean Medicine, Wonkwang University, Iksan 54538, Republic of Korea; baegs888@wku.ac.kr; 4 Research Center of Traditional Korean Medicine, Wonkwang University, Iksan-daero, Iksan 54538, Republic of Korea

**Keywords:** AVE, ulcerative colitis, dextran sodium sulfate, inflammation, tight junctions

## Abstract

The pathogenesis of inflammatory bowel diseases (IBD), such as ulcerative colitis and Crohn’s disease, remains incompletely understood. *Amomum villosum* Lour. (Zingiberaceae) is a traditional herbal medicine used across Asia to treat digestive and inflammatory disorders. This study investigated the therapeutic effects of a water extract derived from the fruits of AV (referred to as AVE) in a mouse model of colitis induced by dextran sulfate sodium (DSS). The protective effects of AVE were evaluated by monitoring changes in body weight and colon length, as well as histological and molecular markers of inflammation. Neutrophil infiltration and levels of inflammatory cytokines in colon tissue and serum were assessed, and the integrity of the intestinal epithelial barrier was examined via Western blot analysis. Treatment with AVE significantly alleviated DSS-induced colitis, as evidenced by improved body weight, longer colon length, and reduced inflammatory responses. AVE administration restored tight junction protein expression (zonula occludens-1 [ZO-1] and occludin), suppressed phosphorylation of mitogen-activated protein kinases—specifically, extracellular signal-regulated kinase (ERK) and p38—and inhibited the expression of inflammatory mediators including tumor necrosis factor-alpha (TNF-α), cyclooxygenase-2 (COX-2), interleukin (IL)-6, IL-1β, and myeloperoxidase (MPO) activity. These findings suggest that oral AVE treatment effectively protects against experimental colitis by modulating inflammatory signaling and preserving epithelial barrier integrity. Further studies are warranted to explore the clinical potential and safety of AVE in the management of IBD.

## 1. Introduction

The two most prevalent forms of inflammatory bowel disease (IBD) are ulcerative colitis and Crohn’s disease, which are complicated, multifactorial conditions with unclear etiologies [1]. Many mouse models of colitis have been produced over the past 20 years to research human IBD mechanistically [2]. These animal models are essential resources for understanding the underlying mechanisms of IBD etiology and for assessing various treatment options. Because of its simplicity and many similarities to clinical ulcerative colitis, the DSS-induced colitis model is among the best models for chemically induced colitis in rodents. When this paradigm is used, its benefits and drawbacks must be considered. With an emphasis on both the specific procedure and variables that may have an impact on DSS-induced pathology, the current protocol attempted to provide a thorough description of the DSS-induced colitis model.

Because the mammalian gastrointestinal tract is constantly exposed to various germs, toxins from food, and environmental factors, it is extremely susceptible to illness. Two basic inflammatory bowel diseases (IBDs) of the gastrointestinal tract are Crohn’s disease (CD) and ulcerative colitis (UC). Both conditions are characterized by acute and chronic intestinal inflammation with a complex etiology [3]. Many animal models have been created in the past few decades to assess prospective human treatments, characterize the intricacy of IBD pathogenesis, and identify underlying molecular pathways [4].

Dextran sodium sulfate (DSS) is a pharmacologic colitogen with anticoagulant characteristics that is likely the most commonly employed mouse model of colitis to generate illness. DSS is a negatively charged, water-soluble sulfated polysaccharide that has a very fluctuating molecular weight that can be between 5 and 1400 kDa. Drinking water containing 40–50 kDa DSS causes colitis in rodents in parallel with UC in humans [5]. While the exact mechanism by which DSS induces intestinal inflammation is not fully understood, it is believed that DSS causes damage to the epithelial monolayer of the large intestine, allowing proinflammatory substances such as bacteria and their byproducts to infiltrate the surrounding tissue, leading to inflammation. Because of its speed, ease of use, controllability, and repeatability, the DSS-induced colitis model is highly useful in IBD research. Changes in the DSS concentration and frequency of injection can produce types of intestinal inflammation that are acute, chronic, and relapsing.

Fruits of *Amomum villosum* Lour. (AV) (Zingiberaceae) is the botanical source of the herbal extract referred to as AV water exrtract (AVE). Recent studies have demonstrated that extracts from various parts of AV, including its fruits, stems, and leaves, exhibit notable anti-inflammatory and antioxidant properties. For example, phenolic and terpenoid compounds isolated from the fruits of AV were shown to suppress inflammatory responses by modulating key signaling pathways such as MAPK and NF-κB [6]. Similarly, stem and leaf extracts have been reported to exert anti-inflammatory and radical-scavenging effects in vitro and in vivo [7]. In addition, AV extract has shown therapeutic efficacy in DSS-induced colitis models by alleviating histological damage and inflammatory cytokine expression [8]. Beyond its anti-inflammatory properties, AVE has also been reported to significantly reduce postprandial glucose and insulin secretion in healthy individuals [9], and in mouse models of high-fat and high-cholesterol diet-induced obesity [10,11]. Furthermore, AVE markedly inhibits α-glucosidase activity, supporting its potential as a multifunctional bioactive agent [12].

Recent phytochemical investigations of *A. villosum* fruits have identified a range of active constituents such as vanillic acid, bornyl acetate, and other phenolic and terpenoid compounds, which are associated with modulation of inflammatory signaling pathways, including MAPK and NF-κB [6,7]. In particular, vanillic acid, one of the major phenolic components of *A. villosum* fruit, has been demonstrated to restore intestinal epithelial integrity and suppress proinflammatory cytokines in colitis models [13]. These findings suggest that AVE contains bioactive molecules with therapeutic relevance for IBD.

In the present study, we focused on the effects of AVE on experimental colitis. We aimed to evaluate its anti-inflammatory and intestinal barrier-protective effects in a DSS-induced colitis mouse model. To this end, we assessed changes in body weight, colon length, myeloperoxidase (MPO) activity, inflammatory cytokine levels (IL-6, IL-1β, TNF-α), phosphorylation of MAPK pathway proteins (ERK, p38), and expression of tight junction markers (ZO-1, occludin). Furthermore, we sought to clarify the underlying molecular mechanisms, with a particular focus on the involvement of MAPK signaling and tight junction regulation.

## 2. Materials and Methods

### 2.1. Preparation of AVE

Fruits of *AV* were used as the biological material for extract preparation. A total of 100 g of finely powdered fruit was extracted in 1 L of distilled water at 100 °C for 3 h using a reflux extraction apparatus. After cooling to room temperature, the extract was filtered through a 0.45 µm membrane to remove particulate matter. The filtrate was then concentrated under reduced pressure using a rotary evaporator and subsequently freeze-dried to obtain a dry powder. The yield of the freeze-dried extract was approximately 9.25 g, corresponding to a 9.25% extraction yield based on the initial dry weight of the fruit powder. The obtained extract was brown in color, hygroscopic, and readily soluble in aqueous media. It was stored at −20 °C in an airtight container until used in subsequent experiments. This preparation was referred to as AVE and used consistently throughout all experiments.

### 2.2. Dextran Sodium Sulfate (DSS) Model of Mouse Colitis

Eight-week-old male C57BL/6J mice weighing 20 ± 2 g were acquired from Orient Bio, Inc., in Seongnam, Korea. The mice were acclimated for one week while having unlimited access to tap water and regular mouse food. Eight animals were kept in cages with a 12:12 h light cycle, a regulated temperature of 23 °C, and a humidity of 55 ± 10%. The Institutional Animal Care and Use Committee (IACUC, permit number: WKU23-1) provided clearance for all research to be carried out. Six groups of animals (n = 8 per group) were randomly assigned to assess the pharmacological effects of AVE on ulcerative colitis: control, DSS (2.5% dextran sulfate sodium, negative control), DSS and low-dose AVE (100 mg/kg body weight; DSS+AVE), DSS and medium-dose AVE (200 mg/kg; DSS+AVE), DSS and high-dose AVE (500 mg/kg; DSS+AVE), and DSS and 5-aminosalicylic acid (5-ASA) (75 mg/kg; DSS+5-ASA; positive control). Over the course of 20 days, the mice were given AVE or 5-ASA in 200 μL of PBS (1X) every day via an intragastric cannula.

The DSS-induced colitis model was developed in accordance with previously mentioned procedures [11]. In brief, colitis was induced by adding DSS solution (molecular weight = 36–50 kDa; MP Biomedicals, Santa Ana, CA, USA) to the drinking water at a concentration of 2.5% over a period of 6 days (from days 15–20). Every day, a new DSS solution was made. The daily variations in body weight were noted. All the mice were anesthetized with isoflurane on day 21 of the experiment; blood was drawn, and the serum was separated via centrifugation at 600× *g* for 10 min at 4 °C to measure the serum IL-6 level. Finally, all of the animals were killed via cervical dislocation. Prior to sacrifice, all of the mice had fasted for at least 8 h. The intestinal contents were removed and washed with sterile PBS (1X) after the colon lengths were measured. The mouse colonic segments were divided into 1 cm slices and kept at −80 °C until needed for additional tests.

### 2.3. MPO Assay

Myeloperoxidase (MPO) activity in colon tissue was measured as an index of neutrophil infiltration. After sacrifice, a defined segment of the distal colon (~1 cm) was excised, rinsed with cold PBS to remove fecal material, and weighed. The tissue was then homogenized in ice-cold 50 mM phosphate buffer (pH 6.0) containing 0.5% hexadecyltrimethylammonium bromide (HTAB) using a mechanical tissue homogenizer. The homogenates were sonicated briefly and centrifuged at 13,000× *g* for 15 min at 4 °C. Supernatants were collected for MPO analysis.

For the MPO assay, 10 µL of each supernatant sample was incubated with 90 µL of a reaction buffer containing 50 mM phosphate buffer, 0.167 mg/mL o-dianisidine dihydrochloride, and 0.0005% hydrogen peroxide. The change in absorbance was measured at 460 nm over 5 min using a microplate reader. MPO activity was calculated based on a standard curve generated from purified MPO enzyme (Sigma-Aldrich, St. Louis, MO, USA) and normalized to tissue protein content, which was determined using a BCA protein assay kit (Sigma-Aldrich, St. Louis, MO, USA). The results are expressed as units of MPO activity per mg of protein (U/mg protein).

### 2.4. Enzyme-Linked Immunosorbent Assay (ELISA)

Blood samples were allowed to clot at room temperature for 30 min and then were separated by centrifugation at 600× *g* for 10 min at 4 °C and stored at −80 °C until analysis. For colon tissue, approximately 100 mg of distal colon was excised, rinsed with cold PBS, and homogenized in 1 mL of cold PBS using a mechanical homogenizer. The homogenates were centrifuged at 13,000× *g* for 15 min at 4 °C, and the resulting supernatants were collected and stored at −80 °C. Then, the levels of IL-6 and IL-1β in the serum and colon tissue were determined via enzyme-linked immunosorbent assay (ELISA). The 96-well ELISA plate was properly labeled. Then, 100 μL of primary antibody (Invitrogen, Carlsbad, CA, USA) was added to each well. The plate was sealed with plate sealer and left overnight at 4 °C with gentle shaking. After the solution was discarded, the wells were washed twice with 1X Wash Buffer (300 μL each). Each well was filled with 200 μL of blocking buffer. After the plate was sealed with a plate sealer, it was incubated for 1~2 h at room temperature. The wells were washed four times with 1X Wash Buffer (300 μL each) after the solution was discarded. To each well, 50 μL of the sample or the standard (BD Biosciences, San Jose, CA, USA) was added. After the plate was covered with a plate sealer, it was incubated for 3 h at room temperature with gentle shaking. The solution was discarded, and the wells were washed four times with 1X wash buffer (300 μL each). To each well, 50 μL of secondary antibody (Invitrogen, CA, USA) was added. After the plate was covered with a plate sealer, it was incubated for 50 min at room temperature with gentle shaking. After the solution was discarded, the wells were washed four times with 1X wash buffer (300 μL each). Fifty microliters of diluted streptavidin-HRP (Invitrogen, CA, USA) reagent were added to each well. The plate was sealed with a plate sealer and aluminum foil before being incubated for 30 min at room temperature with light shaking. The wells were washed six times with 1X Wash Buffer (300 μL each) after the solution was discarded. To each well, 50 μL of TMB (Invitrogen, CA, USA) was added. The plate was covered with a plate sealer and aluminum foil and incubated for five minutes at room temperature while being gently shaken. To halt the reaction, 50 mL of Stop Solution was added to each well. The absorbance was read at 450 nm.

### 2.5. Real-Time Reverse Transcription-Polymerase Chain Reaction (RT-PCR)

To analyze the mRNA levels, colon tissue from the mice was used. The mRNA expression levels of TNF-α, ZO-1, and occludin were determined via reverse transcription polymerase chain reaction. TRIzol reagent (Invitrogen, Carlsbad, CA, USA) was used to extract total colon RNA in accordance with the manufacturer’s instructions. After that, a PrimeScriptTM qPCR RT kit (TaKaRa Bio, Kusatsu, Japan) was used to reverse transcribe 2000 ng of total RNA to cDNA. A real-time PCR instrument (Applied Biosystems, Foster city, CA, USA) and SYBR Green PCR master mix were used for qPCR to measure the target genes’ mRNA expression levels. The degrees of gene expression for each sample were normalized relative to β-actin gene expression with the 2^−ΔΔCt^ method. A list of the primer sequences can be found in Table 1.

### 2.6. Western Blotting

In RIPA lysis buffer (Thermo Fisher Scientific, Waltham, MA, USA), the colonic tissue was homogenized via an ice bucket. A BCA protein quantification kit (Thermo Fisher Scientific, CA, USA) was used to measure the protein quantity in the collected supernatant after centrifugation (16,000× *g*, 10 min). The supernatant samples were subjected to sodium dodecyl sulfate‒polyacrylamide gel electrophoresis (SDS‒PAGE; 8% or 10% separation gel and 5% stacking gel) after being heated at 95 °C for 10 min with 5X loading buffer (Elpis Biotech, Daejeon, Republic of Korea). Polyvinylidene fluoride (PVDF) membranes were used to transfer the gels (GE Healthcare Life Sciences, Buckinghamshire, UK). Primary antibodies were added to the membranes, which were incubated overnight at 4 °C after they were blocked with 5% BSA (Sigma-Aldrich, MO, USA) or skim milk powder (BD Difco, Franklin Lakes, NJ, USA) (*w*/*v*) at 4 °C for 2 h. Primary antibodies against p-ERK (1:1000), ERK (1:1000), phosphorylated p38 MAPK (1:1000), and p38 MAPK (1:1000) were obtained from Cell Signaling Technology (Danvers, MA, USA). Santa Cruz Technology (Santa Cruz, CA, USA) provided anti-COX-2 (1:1000), anti-β-actin (1:1000), anti-ZO-1 (1:1000), and anti-occludin (1:1000) antibodies. Abcam (Cambridge, UK) provided the TNF-α (1:1000) primary antibody. The next day, the membranes were thoroughly washed with 1X TBST before being incubated with HRP-conjugated secondary antibodies for 1 h at 4 °C. Goat anti-mouse IgG H&L (HRP) (1:1000), goat anti-rabbit IgG H&L (HRP) (1:1000), and goat anti-rat IgG H&L (HRP) (1:1000) secondary antibodies were purchased from Abcam (Cambridge, UK). The protein bands were captured by imaging equipment (AE-9300 Ez-capture MG/AE-9160 Ez-capture ST, Tokyo, Japan) after the membrane had been subjected to enhanced chemiluminescence (ECL) detection reagent (1:1) (Millipore, Darmstadt, Germany). Densitometric studies of p-ERK, ERK, phospho-p38 MAPK, p38 MAPK, COX-2, ZO-1, occludin, and TNF-α were standardized via internal standards of β-actin.

### 2.7. High Liquid Chromatography (HPLC) Analysis

Standardized vanillic acid (cat. no. 94770) was acquired from Sigma-Aldrich (St. Louis, MO, USA). 50% Methanol was used to dissolve the extract and prepare the standard. High-performance liquid chromatography (HPLC) was conducted on an Agilent HPLC system (Santa Clara, CA, USA). The extract and standard were investigated under the following conditions: column was Waters Sunfire C18 (250 mm × 4.6 mm, 5 μm; Waters, Milford, MA, USA) and the mobile phase consisted of distilled water containing 0.1% Trifluoroacetic acid (solvent system A) and methanol (solvent system B) in a gradient mode. The gradient program was used as follows: A/B (80:20, *v*/*v*) from 0 to 20 min, linear change from A/B (70:30, *v*/*v*) to A/B (50:50, *v*/*v*) from 20 to 40 min, A/B (1:90, *v*/*v*) from 45 min to 50 min, linear change from A/B (10:90, *v*/*v*) to A/B (80:20, *v*/*v*) from 50 min to 55 min. The sample injection volume was 10 µL, flow rate was set to 0.7 mL/min, column temperature was set to 35 °C, and UV wavelength was set to 260 nm. The peak was identified based on retention time and comparison with the authentic reference compound injected.

### 2.8. Calibration Curve of Vanillic Acid

A series of calibration standard solutions of vanillic acid were prepared in methanol. Each concentration was examined thrice. The calibration curve was produced by plotting the peak area under curve versus the concentration of the standard. The regression equation was used to determine the concentration of vanillic acid in samples.

### 2.9. Statistical Analysis

SPSS software package v. 26.0 (IBM, Armonk, NY, USA) was used for statistical analysis with Duncan’s multiple comparison evaluation. The results are presented as the means ± standard deviations (SDs), and a statistically significant value of *p* < 0.05 was used to determine the results.

## 3. Results

### 3.1. The Administration of AVE Reduces the Clinical Manifestations and Disease Activity of DSS-Induced Colitis in Mice

**Figure 1:**
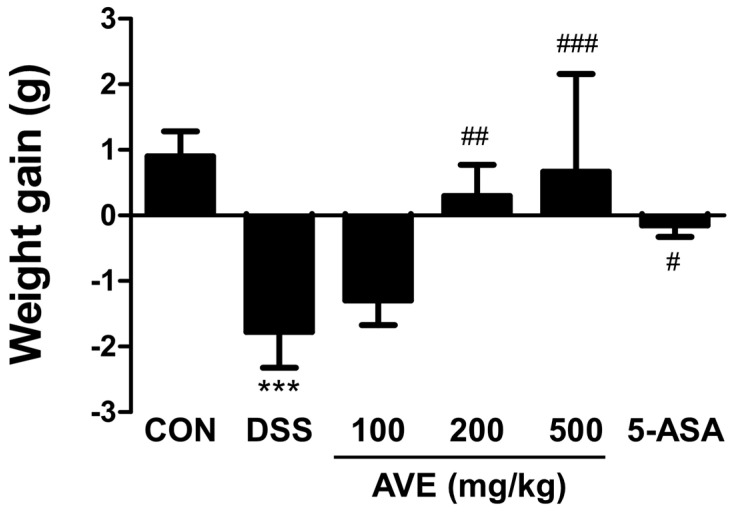
Effects of *Amomum villosum* extract (AVE) on the body weights of DSS-induced mice. Mice were orally administered AVE (100, 200, or 500 mg/kg) or 5-ASA (75 mg/kg) once daily for 20 days. DSS (2.5%) was administered in drinking water from days 15 to 20 to induce colitis. Body weights were monitored daily to assess the impact of DSS-induced colitis and the therapeutic effects of AVE. DSS treatment caused significant weight loss, which was attenuated by AVE in a dose-dependent manner. Data are presented as means ± SD. Statistical significance is indicated as *** *p* < 0.001 vs. the CON group, # *p* < 0.05, ## *p* < 0.01, and ### *p* < 0.001 vs. the DSS group. n = 8 per group.

**Figure 2:**
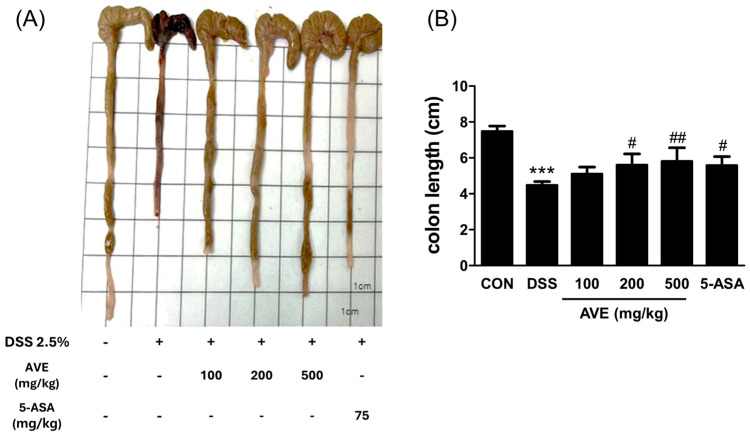
Effect of *Amomum villosum* extract (AVE) on colon length in DSS-induced mice. Mice were orally administered AVE (100, 200, or 500 mg/kg) or 5-ASA (75 mg/kg) once daily for 20 days. DSS (2.5%) was administered in drinking water from days 15 to 20 to induce colitis. Colon length was measured post sacrifice to evaluate the severity of colitis. (**A**) Colon length and (**B**) Semi-quantification of colon length from (**A**). DSS treatment significantly shortened the colon length, and this effect was dose-dependently alleviated by AVE. This figure complements the body weight changes shown in Figure 1, illustrating that AVE not only preserves body weight but also mitigates physical damage to the colon. Data are presented as means ± SD. Statistical significance is indicated as *** *p* < 0.001 vs. the CON group, # *p* < 0.05, and ## *p* < 0.01, vs. the DSS group. n = 8 per group.

According to the current findings, mice given drinking water enriched with 2.5% DSS for six days presented symptoms similar to those of inflammatory bowel disease (IBD), such as bleeding in the rectal cavity, diarrhea, a shortened colon, a loss of body weight, bent back, raised hair, sepsis symptoms, and decreased movement. The DSS-treated mice continued to lose body weight, as shown in Figure 1. In a dose-dependent way, the AVEs hampered weight reduction (Figure 1). Compared with those of the control group, the length of the colon was considerably reduced as a result of DSS treatment, which was subsequently mitigated by AVE administration in a dose-dependent manner (Figure 2).

### 3.2. Impact of the AVE on MPO Regulation

**Figure 3:**
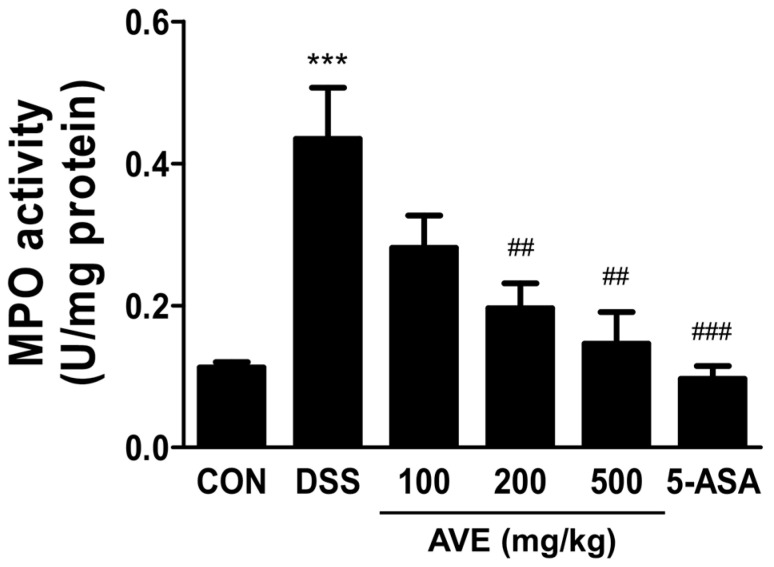
Effect of *Amomum villosum* extract (AVE) on MPO activity in the colon tissue of DSS-induced mice. Mice were orally administered AVE (100, 200, or 500 mg/kg) or 5-ASA (75 mg/kg) once daily for 20 days. DSS (2.5%) was administered in drinking water from days 15 to 20 to induce colitis. The activity of myeloperoxidase (MPO), a marker of neutrophil infiltration and inflammation, was significantly increased by DSS treatment and dose-dependently reduced by AVE. Data are presented as means ± SD. Statistical significance is indicated as *** *p* < 0.001 vs. the CON group, ## *p* < 0.01, ### *p* < 0.001 vs. the DSS group. n = 8 per group.

As shown in Figure 3, the colons of the mice in the DSS group had considerably greater MPO activity than those of the control group did (*p* < 0.001), and this activity dramatically decreased (*p* < 0.01) in a dose-dependent manner with 100−500 mg/kg AVE.

### 3.3. Influences of AVE on Inflammatory Cytokine Regulation

**Figure 4:**
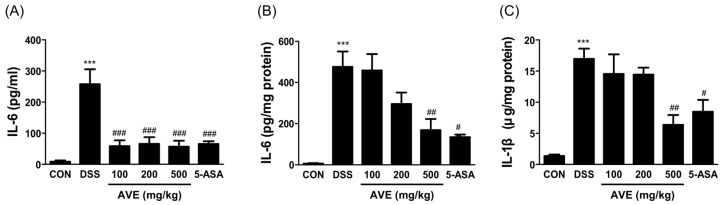
Effects of *Amomum villosum* extract (AVE) on cytokine levels in the serum (**A**) and colon tissue (**B**,**C**) of DSS-induced mice. Mice were orally administered AVE (100, 200, or 500 mg/kg) or 5-ASA (75 mg/kg) once daily for 20 days. DSS (2.5%) was administered in drinking water from days 15 to 20 to induce colitis. The levels of IL-6 and IL-1β were measured to assess the inflammatory response. DSS treatment markedly increased the levels of these cytokines in both serum and colon tissue, whereas AVE treatment significantly suppressed this increase, demonstrating its anti-inflammatory potential. Data are presented as means ± SD. Statistical significance is indicated as *** *p* < 0.001 vs. the CON group, # *p* < 0.05, ## *p* < 0.01 vs. the DSS group. n = 8 per group.

As shown in Figure 4A, treatment with DSS markedly elevated serum IL-6 expression (*p* < 0.001), whereas treatment with AVE and 5-aminosalicylic acid markedly suppressed the aberrant increase in IL-6 (*p* < 0.05). The mice in the DSS group had significantly higher colon IL-6 and IL-1β levels (*p* < 0.001), as shown in Figure 4B,C. Following the administration of 100−500 mg/kg AVE, a significant decrease (*p* < 0.01) in the contents of IL-6 and IL-1β was detected.

### 3.4. The Effect of AVE on Gene and Protein Expression Linked to Inflammation

**Figure 5:**
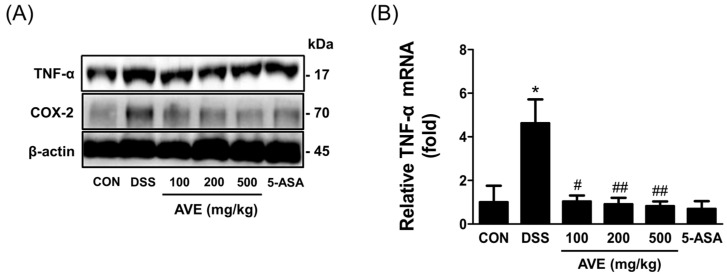
Effects of *Amomum villosum* extract (AVE) on colon inflammation-related protein (**A**) and mRNA expression (**B**) in DSS-induced mice. Mice were orally administered AVE (100, 200, or 500 mg/kg) or 5-ASA (75 mg/kg) once daily for 20 days. DSS (2.5%) was administered in drinking water from days 15 to 20 to induce colitis. DSS treatment led to significant upregulation of these inflammatory markers, which were significantly downregulated by AVE treatment. This figure illustrates the capacity of AVE to modulate key inflammatory mediators in colitis. Data are presented as means ± SD. Statistical significance is indicated as * *p* < 0.05 vs. the CON group, # *p* < 0.05, ## *p* < 0.01 vs. the DSS group. n = 8 per group.

Compared with those in the control group, the expression levels of TNF-α (both mRNA and protein) and COX-2 (protein) in the DSS control group were significantly greater. Our results demonstrated that treatment with AVE led to a significant reduction in the expression of COX-2 (protein) and TNF-α (mRNA and protein). Specifically, AVE treatment downregulated TNF-α mRNA levels by approximately 3-fold (*p* < 0.05), while the protein levels of TNF-α and COX-2 were reduced to levels similar to those observed in the control group, as illustrated in Figure 5.

### 3.5. The Impact of AVE on Tight Junction mRNA and Protein Regulation

**Figure 6:**
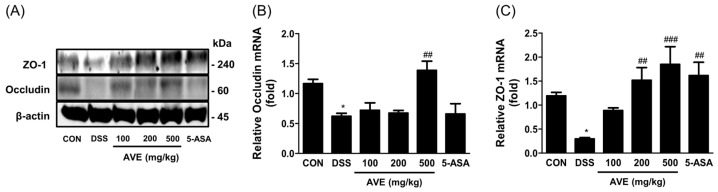
Effects of *Amomum villosum* extract (AVE) on intestinal tight junction protein (**A**) and mRNA expression levels (**B**,**C**) in DSS-induced mice. Mice were orally administered AVE (100, 200, or 500 mg/kg) or 5-ASA (75 mg/kg) once daily for 20 days. DSS (2.5%) was administered in drinking water from days 15 to 20 to induce colitis. The expression of ZO-1 and occludin, crucial components of the intestinal barrier, was disrupted by DSS treatment. AVE administration restored the expression of these proteins, suggesting its protective role in maintaining intestinal barrier integrity. Data are presented as means ± SD. Statistical significance is indicated as * *p* < 0.05 vs. the CON group, ## *p* < 0.01 and ### *p* < 0.001 vs. the DSS group. n = 8 per group.

Figure 6 shows that following DSS treatment, ZO-1 and occludin mRNA and protein expression significantly decreased (*p* < 0.05) compared with that in the control group. The mRNA and protein expression levels of occludin and ZO-1 were dramatically increased (*p* < 0.01) by AVE treatment.

### 3.6. The Impact of AVE on MAPK Signaling Regulation

**Figure 7:**
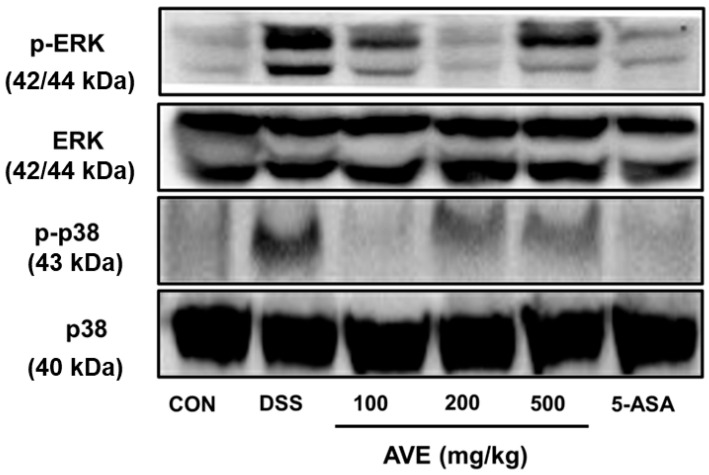
Effects of *Amomum villosum* extract (AVE) on MAPK signaling (P38/ERK) protein expression levels in DSS-induced mice. Mice were orally administered AVE (100, 200, or 500 mg/kg) or 5-ASA (75 mg/kg) once daily for 20 days. DSS (2.5%) was administered in drinking water from days 15 to 20 to induce colitis. The phosphorylation levels of p38 and ERK were evaluated to explore the involvement of MAPK signaling in the anti-inflammatory effects of AVE. DSS treatment increased the phosphorylation of these proteins, whereas AVE treatment significantly inhibited this activation, indicating its suppression of the MAPK pathway. n = 8 per group.

Figure 7 shows that, compared with those in the normal control group, the phosphorylation levels of p38 and ERK in the DSS group were considerably greater (*p* < 0.05). AVE treatment significantly inhibited the phosphorylation of these MAPK pathways, increasing their levels to levels closer to those of the normal control group, indicating a potential mechanism by which AVE exerts its anti-inflammatory effects.

### 3.7. Identification of Vanillic Acid in AVE

**Figure 8:**
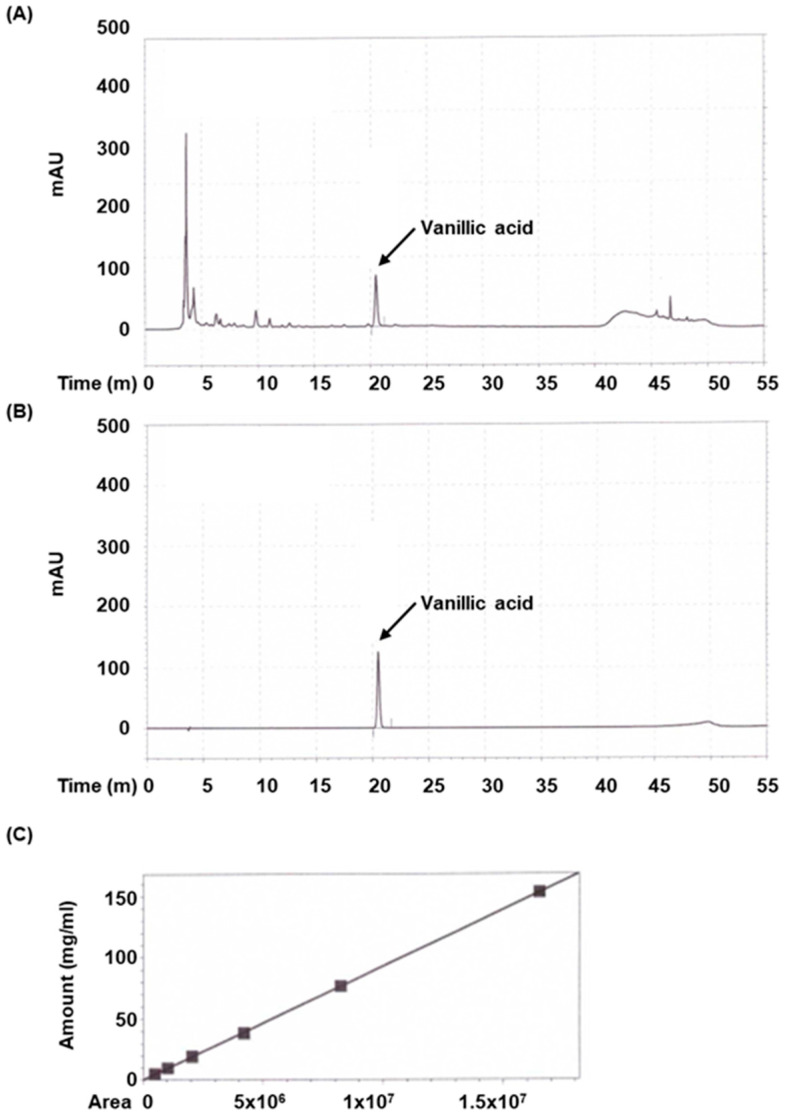
High-performance liquid chromatography (HPLC) analysis of *Amomum villosum* extract (AVE). Chromatograms of (**A**) AVE and (**B**) vanillic acid as standards at 260 nm. (**C**) Calibration curve of vanillic acid.

Next, we decided to identify the component contributing to the anti-inflammatory activity of AVE using the HPLC. As shown in Figure 8, HPLC analysis of AVE exerted a major peak (around 20 min), which was identified as vanillic acid. The concentration of vanillic acid was calculated by using calibration curve (R^2^ = 0.999963, y = 9.26444 × 10^−6^x − 0.0363543). It was ascertained that AVE contained approximately 1.09 ± 0.01 mg/g of vanillic acid.

## 4. Discussion

Over the past two decades, our understanding of human inflammatory bowel disease (IBD), including ulcerative colitis (UC) and Crohn’s disease (CD), has greatly improved, in large part due to the development of well-characterized experimental models in rodents [14]. Among these, the dextran sulfate sodium (DSS)-induced murine colitis model has played a pivotal role by providing a reproducible and clinically relevant platform for evaluating potential therapeutic agents and dissecting disease mechanisms [14,15]. This model mimics many features of human UC, such as mucosal ulceration, epithelial barrier disruption, and innate immune activation, thereby offering valuable insights into the pathophysiology of IBD. In the present study, we aimed to further elucidate the molecular mechanisms by which AVE exerts its therapeutic effects in DSS-induced colitis. Consistent with clinical improvement, AVE treatment significantly attenuated weight loss and colon shortening in DSS-treated mice. These results indicate that AVE effectively mitigates the clinical manifestations of colitis.

While previous studies have reported the anti-inflammatory properties of AVE in various disease contexts [6,7,8], its specific effects on intestinal inflammation and barrier integrity remained unclear. Our findings demonstrate that AVE downregulates key pro-inflammatory cytokines, including IL-1β, IL-6, and TNF-α, and suppresses activation of the MAPK signaling pathway—particularly the phosphorylation of ERK and p38. Additionally, AVE restores the expression of tight junction proteins (ZO-1 and occludin), thereby strengthening intestinal barrier function. These data collectively suggest that AVE exerts protective effects in colitis through both immunomodulatory and barrier-preserving mechanisms.

According to previous report [16], UC-induced neutrophil infection results in tissue injury, the death of epithelial cells, and barrier degradation. MPO is an enzyme that is a marker of acute inflammation in neutrophils and indicates the extent of neutrophil infiltration [16,17]. Similar to the current findings, earlier studies have demonstrated that natural products reduce MPO activity to treat colitis [18,19]. MPO activity was greater following DSS treatment and decreased in a dose-dependent manner with AVE therapy.

UC produces a large number of inflammatory substances as a result of mast cell and T-cell activation [20]. By stopping the production of TNF-α, IL-6, and IL-1β in animals with DSS-induced colitis, AVE was shown to have anti-inflammatory effects in this study. One important extracellular signal transduction pathway that is triggered by inflammatory mediators and aids in controlling the synthesis of inflammatory cytokines is the MAPK pathway [21]. Cellular processes such as growth, differentiation, survival, and death are regulated by MAPK signaling. ERK, JNK, and p38 are the three main subtypes of MAPKs found in mammals. These proteins phosphorylate downstream substrates, such as transcription factors involved in signal response [22,23]. We assessed the effectiveness of AVE against p38 and ERK, two distinct MAPKs, in this investigation. In the animal model used in this work, DSS phosphorylated p38 and ERK, whereas AVE blocked this phosphorylation. These outcomes are in line with earlier research showing that natural products reduce inflammation via p38 phosphorylation and ERK inhibition [22,23,24].

Intestinal tight junction (TJ) barrier disruption is also caused by the overexpression of TNF-α, IL-1β, and IL-6 [1,2]. Two essential components of the intestinal mucosal barrier are intestinal epithelial cells and tight junction proteins [25]. Intracellular proteins such as ZOs and actin, transmembrane proteins such as ZOs, and adhesion molecules such as occludin and claudin make up tight junction proteins. The integrity and intestinal permeability of the mucosal barrier are ultimately maintained by preventing immune cell activation and aberrant immunological responses of the intestinal mucosa [16,26]. ZO-1 and occludin tight junction protein expression increased in UC model mice treated with AVE. These findings indicate that by increasing the expression of tight junction proteins, AVE may have a protective effect on the intestinal mucosal barrier, which could account for its favorable effect on DSS-induced colitis.

Our results are in line with those of previous report [18], who reported the therapeutic efficacy of AV fruit extract in a rat model of colitis. While both studies used crude water extracts of AV fruits, there are several methodological differences worth noting. First, our study employed a mouse model using dextran sulfate sodium (DSS) to induce colitis, whereas Chen et al. used the 2,4,6-trinitrobenzenesulfonic acid (TNBS)-induced colitis model in rats [18]. These two models differ mechanistically: DSS primarily induces epithelial barrier dysfunction and innate immune activation, while TNBS causes transmural inflammation resembling aspects of Crohn’s disease and involves both innate and adaptive immunity [27]. Second, the extraction protocol in our study involved a longer decoction time (3 h at 100 °C under reflux) compared to the shorter extraction time in Chen’s study, which may influence the concentration and spectrum of bioactive constituents. Despite these differences, both studies consistently demonstrated significant anti-inflammatory effects of AV extract, suggesting that its bioactivity is robust across species, extraction conditions, and experimental colitis models. These findings collectively strengthen the translational potential of AV as a therapeutic agent for IBD.

Recent studies have highlighted the therapeutic potential of vanillic acid, a phenolic compound found in various medicinal plants, in alleviating colitis and other inflammatory conditions. Notably, vanillic acid was shown to restore intestinal epithelial homeostasis in DSS-induced colitis by inhibiting CA9/STIM1-mediated ferroptosis, thereby preventing epithelial cell death and preserving barrier integrity [28]. Additionally, vanillic acid has demonstrated anti-inflammatory and tissue-protective effects in models of ulcerative colitis, reducing histological damage and inflammatory cytokine levels [13]. Beyond its role in colitis model, vanillic acid also exerts modulatory effects on key intracellular signaling pathways, including MAPK and PI3K/AKT/NF-κB, which are critically involved in inflammation and tissue degeneration [29]. In our study, vanillic acid was identified as a major component of AVE, suggesting that the observed protective effects of AVE against DSS-induced colitis may be, at least in part, mediated through vanillic acid’s multifaceted actions on inflammatory signaling pathways. These findings collectively support the therapeutic relevance of AVE and its bioactive constituents in managing colitis.

Given these pharmacological properties and its prominent presence in AVE, vanillic acid was selected as a representative bioactive marker compound for quantitative analysis. This decision was supported by both the literature evidence and our HPLC profiling results, which revealed a distinct peak corresponding to vanillic acid, indicating its abundance in AVE. As the primary aim of this study was to evaluate the therapeutic efficacy of AVE in colitis, vanillic acid served as a rational marker to establish the correlation between phytochemical content and biological activity. Nonetheless, we acknowledge that comprehensive phytochemical profiling—including other known constituents such as bornyl acetate, methyl cinnamate, and various flavonoids—would further enhance our understanding of AVE’s pharmacological effects. Future studies will therefore employ advanced analytical techniques such as LC–MS/MS to identify and quantify additional bioactive compounds.

## 5. Conclusions

In summary, AVE effectively modulates the aberrant immune response in the intestinal mucosa, strengthens intestinal barrier function, and inhibits the production of inflammatory mediators by blocking MAPK pathways under experimental conditions. These findings suggest that AVE holds promise as a potential therapeutic agent for ulcerative colitis (UC). However, further studies, particularly well-designed clinical trials, are essential to confirm these results and determine the therapeutic efficacy and safety of AVE in broader clinical settings. Additionally, future research should aim to elucidate the precise molecular mechanisms of AVE and explore its potential synergistic effects when used in combination with current UC therapies.

## Figures and Tables

**Table 1:** Sequences of primers used for real-time polymerase chain reaction (PCR).

Gene	Prime Sequence (5′ → 3′)
β-actin	Forward: ATCACTATTGGCAACGAGCG Reverse: TCAGCAATGCCTGGGTACAT
TNF-α	Forward: TACCTTGTCTACTCCCAGGTTCTCT Reverse: GTGTGGGTGAGGAGCACGTA
ZO-1	Forward: GGGGCCTACACTGATCAAGA Reverse: TGGAGATGAGGCTTCTGCTT
Occludin	Forward: CACACTTGCTTGGGACAGAG Reverse: TAGCCATAGCCTCCATAGCC

## Data Availability

The data used to support the funding of this study are available from the corresponding author upon request.
